# Full shuffle and p-rectified semi-inner powerful IoU-based chili pepper flower recognition with environment-aware YOLO11

**DOI:** 10.3389/fpls.2026.1935123

**Published:** 2026-09-16

**Authors:** Cui-Ping Zhang, Zhi-Yong Wang, Xue-Wei Wang, Zhi Li

**Affiliations:** 1 School of Artificial Intelligence, Weifang University of Science and Technology, Weifang, Shandong, China; 2 School of Information Science and Engineering, Weifang University of Science and Technology, Weifang, Shandong, China; 3 Academic Affairs Department, Sichuan Technology and Business University, Chengdu, Sichuan, China

**Keywords:** agriculture image analysis, deep learning, flower detection, smart agriculture, YOLO

## Abstract

As a common greenhouse-grown commercial crop, chili pepper yields have always been a focus of attention. Effective management is essential for both environmental sustainability and high productivity in greenhouses. Whether for growth monitoring, yield estimates, and automated production, precise identification of chili flowers is critical. To this goal, this paper works upon the YOLO11 object detection model, focusing on the precise detection of chili flowers. The diversity of samples across various scenarios is increased by utilizing a self-built dataset of chili flowers in greenhouses and integrating data augmentation. To address YOLO11’s limitations in this task, this paper proposes a unified framework that integrates three modules: (1) full shuffle to enhance information exchange between model channels and optimize weights; (2) Since YOLO11 cannot incorporate external environmental data during training, the environment-aware C3k2 is introduced; and (3) The proposed p-rectified Semi-inner Powerful IoU accelerates model convergence and flexibility. Experiments show that our method obtains 84.8% mAP50, 50.2% mAP50-95, 81.1% Precision, and 75.6% Recall, outperforming the baseline YOLO11’s 77.3% by 7.5 percentage points, as well as YOLOv8, YOLO12, RTDETR, and Faster R-CNN under the same experimental conditions. Therefore, in terms of performance, our method generally surpasses existing algorithms for chili flower detection. Research on detecting chili flowers in greenhouses remains limited. The study offers valuable insights for the advancement of smart agriculture. Future work will further explore the model’s generalization capabilities across multiple varieties and growth stages, as well as deploy it on embedded devices for practical application.

## 1 Introduction

As one of the major commercial crops, chili peppers are highly compatible with greenhouse environments’ temperature, light, water, and air control capabilities, with their biological characteristics of being heatloving, moderately light-demanding, having shallow root systems, and being highly sensitive to water and nutrients. As a result, greenhouse cultivation of chili peppers represents a high-value agricultural production system worldwide. To boost yields, in addition to meticulous management of growth factors, artificial pollination must be well-executed, especially in enclosed environments like greenhouses with low air circulation and few insects, as this may dramatically improve fruit set rates. However, due to the small and dense nature of chili flowers, traditional manual pollination is almost impossible in large-scale greenhouses. Conventional methods are labor-intensive and time-consuming, with pollination outcomes entirely dependent on the farmer’s experience—a practice unsustainable in high-density facility agriculture.

Smart agriculture is using disruptive technologies to reconstruct crop management paradigms as conventional agriculture transforms into digitalization. Smart agriculture is a new mode of agricultural production that integrates contemporary information technology to intelligentize the entire chain, including management, operation, processing, storage, transportation, sales, and service. As an important component of agricultural intelligence, monitoring crop growth processes significantly impacts yield prediction and field management. The foundation of automated pollination lies in equipping machines with the ability to perceive and locate their target—the flower. This study builds a chili flower detection algorithm to support agricultural production management in greenhouses.

The calyx diameter of chili flowers is typically 5–8 mm, which is white or light yellow and has low contrast against a green background. This small-scale characteristic is prone to missed detection in routine inspections. Additionally, water droplets easily form in greenhouse environments with high humidity, distorting petal textures. Due to high-density planting, approximately 35% of the flowers are only partially visible, making it challenging for standard algorithms to locate targets reliably. Light reflections on the petal surface form highlight noise, severely interfering with color feature extraction.

The rapid development of AI has brought a glimmer of hope, with deep learning-based image detection shining brightest. There are many types of methods, including ViT, Swin Transformer, and YOLO (You Only Look Once) (Redmon et al., 2016), which is the most representative. YOLO’s single-stage architecture balances the accuracy and speed of recognition, employing feature pyramids and multi-scale prediction mechanisms to improve its capacity to withstand interference in complex agricultural scenarios.

A review of the existing literature reveals that current research primarily focuses on 1) crop detection, including fruits, seeds, branches, leaves, and diseases; The methods employed vary widely, ranging from traditional machine learning approaches such as SVM and Bayesian methods to deep learning algorithms like R-CNN and YOLO. However, flower detection is relatively uncommon. 2) Among the limited research on flower detection, most studies focus on flower detection in open-field crops, while research on chili flower detection in greenhouse environments is relatively rare.

Based on the above analysis, the task of detecting chili flowers in complex greenhouse environments essentially involves examining the combined effects of multiple factors, including high-density plant, small target size (5-8mm), low contrast with leaves and the surrounding environment, approximately 35% partial occlusion, and lighting interference. This study enhances YOLO’s adaptability to chili flower detection circumstances, increases accuracy, and provides data assistance for precision pollination, water and fertilizer management, and other agricultural activities. The main contributions of this paper are as follows:

1. In light of partial shuffle’s drawbacks, we suggest full shuffle to improve feature extraction across various channels without raising computational complexity.
2. Unlike conventional closed-set object detection, we guide the model toward open-set recognition using natural language descriptions. By incorporating textual descriptions of the external environment into the process, we improve the model’s capacity for generalization and resilience to environmental changes.
3. By introducing an improved version of Powerful-IoU, we have reduced computational complexity while improving the model’s convergence speed and flexibility.
4. To ensure data diversity and correctly depict the complexity of real-world greenhouse circumstances, we gathered data and created a dataset in actual greenhouse environments. Our goal is to improve the model’s ability to manage intricate and unpredictable real-world agricultural production scenarios, especially in difficult situations like low light, partial occlusion, or unfavorable shooting angles.

The structure of this article is as follows: Section 2 describes the related work from three perspectives, i.e., developments in crop recognition in greenhouses, YOLO object detection technology, and flower detection. Section 3 details the three key improvements of this study and the enhanced YOLO11 (Khanam and Hussain, 2024) framework. Section 4 discusses the experimental setup, data acquisition and construction, model training, and comparative analysis with YOLO11. Our work is summarized in Section 5.

## 2 Related work

### 2.1 Agricultural vision

Flower recognition in greenhouses has also evolved alongside advancements in machine vision. Early traditional image processing methods combined color, shape, and texture information with machine learning, such as SVM (support vector machines) (Sri and Sridhar, 2025) and Bayesian (Dembla et al., 2025; Arellano et al., 2025) classifiers, to perform basic recognition in stable environments. However, these approaches were susceptible to illumination and occlusion, resulting in a weak generalization ability. Deep learning, such as Faster R-CNN (Rahim and Mineno, 2020) and SSD (Tian et al., 2019), shows significantly superior performance in flower detection for crops like tomatoes (Li and Zhu, 2024), cucumbers (Tang et al., 2024; Xu et al., 2023) and strawberries (Zhou et al., 2023) compared to classic machine learning. However, existing models still have room for improvement in both accuracy and real-time performance under conditions of light fluctuation, dense plant growth, complex backgrounds, and severe occlusions.

Crop detection utilizing computer vision technologies in greenhouses has been a long-term development. Traditional machine vision involves locating regions of interest (ROIs) in input images, extracting feature vectors, predicting object categories within ROIs via region classification and boundary regression, and outputting detection results. SVM (Support vector machines) (Sri and Sridhar, 2025), Bayesian classifiers Gobi and Sharma, 2023), and K-means clustering (Jiang et al., 2023) are the most often used approaches. However, in the complex context of high-density greenhouse cultivation, these methods suffer from low detection accuracy and high computational demands, making real-time detection challenging. Deep learning-based object detection methods are characterized by high detection speeds and accuracy, primarily falling into two categories: region proposal-based methods and regression-based methods. Region proposal-based methods, such as R-CNN, detect in two stages: the first selects candidate regions, and the second employs deep learning methods for classification and regression. In contrast, the regression-based methods, like YOLO, are end-to-end, enabling faster processing speeds suitable for real-time applications. However, it suffers from lower detection accuracy and suboptimal performance.

### 2.2 Small-object detection

In computer vision, small-object detection has always been difficult because of the limited number of target pixels, the absence of feature information, and the downsampling-induced loss of features. Researchers have made significant efforts to address this challenge. We noticed that existing small-object detection methods mainly employ six key technologies to enhance model performance, including multiscale feature fusion, attention mechanism improvement, self-attention mechanisms (Transformers), and data augmentation & sample balancing. Multi-scale feature fusion, exemplified by Feature Pyramid Networks (FPNs) (Lin et al., 2017), is capable of preserving both spatial details and semantic information. The FPN–ISResNet–Faster RCNN model was presented by Hou et al. (2023) for the detection, evaluation, and validation of apple leaf diseases. Experimental results show that this model exhibits high accuracy and generalization capabilities in detecting apple diseases. However, in situations where real-time is crucial, this method necessitates careful evaluation due to its much higher computing needs. Attention mechanisms (Vaswani et al., 2017) and transformers both essentially fall into the category of feature enhancements, as they can strengthen feature representations in small-object regions and suppress irrelevant background noise. Representative attention mechanisms include CBAM (Woo et al., 2018), ECA (Wang et al., 2020), and SA (Zhang and Yang, 2021). Representative transformer architectures include DETR (Carion et al., 2020), VIT (Dosovitskiy et al., 2020), and their variations (Zhu et al., 2020; Zhang et al., 2022; Yao et al., 2021; Srivastava et al., 2025; Siddiqui et al., 2025). Cai et al. (2025) combined attention with C2f DCNv2 in YOLOv8 to adapt to complex traffic scenes, and the novel EMAM was designed to enhance the recognition and representation of small, occluded objects. Experimental results on the KITTI and COCO-traffic datasets showed average accuracy of 97% and 69%, respectively. Miri Rekavandi et al. (2025) gathered over 60 studies from 2020 to 2023 on the application of transformers in small-object detection, along with 12 large-scale datasets spanning various scenarios such as medical, aerial images, and video, to serve as a reference for future research. While attention and transformers offer certain advantages in terms of accuracy, their computational complexity is significantly higher than that of CNNs. Data augmentation and sample balancing are data-wise strategies to mitigate the scarcity of samples for small objects and class imbalance by artificial expansion. These methods include real-time and offline strategies. Real-time strategies include Mosaic, MixUp (Zhang et al., 2023b), and Random Affine, while offline strategies include affine transformations, oversampling, and generative methods (GANs). This kind of method is the simplest to implement but is typically used for preprocessing.

### 2.3 YOLO

YOLOs drive breakthroughs in real-time detection performance via architectural innovation. YOLOv1 (Redmon et al., 2016), proposed by Redmon et al. in 2016, has undergone continuous structural and performance optimization through multiple revisions over nearly a decade, with widespread application across diverse fields. It transforms object detection into a regression challenge, employing neural networks to predict both the category and bounding box coordinates of objects within images. YOLOv2 (Redmon and Farhadi, 2017) builds upon the optimized YOLOv1 by introducing anchor and multi-scale training to enhance small object detection capabilities. YOLOv3 (Redmon and Farhadi, 2018) significantly improves the accuracy by integrating the feature pyramid network (FPN) to better adapt to changes in object sizes. YOLOv5 (Jocher, 2020), a milestone in YOLOs, utilizes CSPDarknet53 as its backbone and integrates Mosaic and Mish, making it the most widely applied object detection algorithm. YOLOv8 (Jocher et al., 2023) replaced v5 due to its significantly improved inference speed, particularly in fine-grained detection within complex scenes. The evolution of YOLO continues unabated. The YOLO12, centered on attention, demonstrates its strong vitality. However, its ecosystem is still underdeveloped, which severely limits application. This study is based on YOLO11 (Khanam and Hussain, 2024), mainly for the following reasons: Firstly, YOLO11 has been available longer than v12, resulting in a more mature ecosystem, which provides convenience for applied research. Secondly, YOLO11 is more advanced than YOLOv1-v10, achieving a relatively balance between accuracy and speed. Finally, most of the current applications are based on the earlier version of YOLO, which provides a broader prospect for our research.

**Table 1** shows a brief history of the YOLO series. A fundamental dilemma is evident in the evolution of YOLO: simplicity means real-time performance, yet complexity leads to high accuracy. Almost every version releases multiple variants (n/s/m/l/x) based on the same architecture to meet the diverse deployment requirements of different scenarios. The differences among these variants primarily lie in their complexity (i.e., depth, width, and input). Generally speaking, the nano and tiny are better suited for agricultural vision tasks due to their small parameter count, low power consumption, and high real-time performance. Bakirci (2025) evaluated the suitability of tiny-, nano-, and small- models for real-time object detection on resource-constrained platforms, further confirming that although the accuracy is lower than that of other variants, this shortcoming can be mitigated through targeted improvements. Therefore, YOLO11n was selected as the baseline.

**Table 1:** Brief history of the YOLO series.

Period	Contribution	Pros	Cons
Foundation [v1(2016)-v3(2018)]	1.Paradigm innovation 2.Anchor boxes+multi-scale	Speed breakthrough	1.Low precision 2.Increased computational cost
Maturity [v4(2020)-v5(2020)]	Engineering and training strategies	Usability leap	Limited academic originality
Optimization [v6(2022)-v7(2022)]	Efficiency and balance	Industrial deployment friendly	Small-target detection still requires tuning
Innovation [v8(2023)-v10(2024)]	Paradigm reconstruction	End-to-end simplicity	Weak performance in dense overlapping scenarios
Latest [v11(2025)-v12(2025)]	Hybrid architecture	Best overall performance	Rebound in computational cost

Notwithstanding these advances, current research shows that the majority of agricultural detection methods still rely on earlier frameworks and fail to fully leverage the latest backbone network structures. Meanwhile, current techniques struggle to tackle the challenges specific to greenhouse environments, such as intricate backgrounds, dense planting, and small target sizes, and are limited in using environmental semantic priors to enhance feature extraction. To address these deficiencies, we provide a unified framework based on YOLO11 that incorporates an environment-aware C3k2 for embedding semantic priors, a full shuffle to enhance information exchange and mitigate overfitting, and an optimized IoU variant for efficient localization.

## 3 Methodology

### 3.1 Full shuffle

Channel shuffle (Zhang and Yang, 2021) employs grouped convolution kernels to shuffle channels, promoting information exchange across them. This approach reduces computational complexity while efficiently extracting features. However, we note that the positions of *A*1 and *C* 3 in **Figure 1a** remain unchanged, meaning that *A*1 and *C* 3 did not participate in the shuffle. In fact, as we can see, the positions of feature maps before and after shuffle can be calculated by Equation 1.

**Figure 1:**
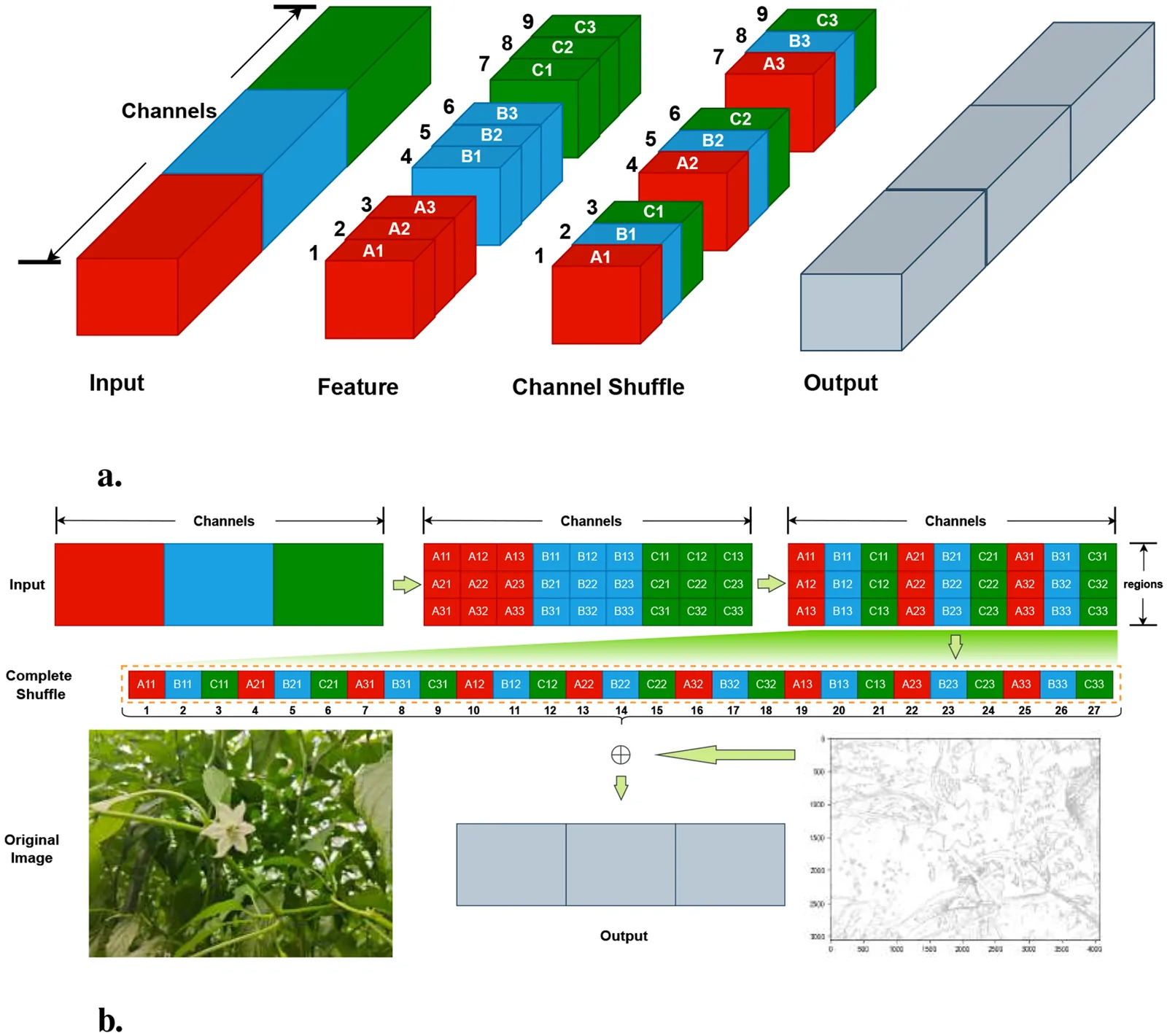
Full shuffle. **(a)** classic shuffle. **(b)** Full shuffle, using # *channels* = # *regions* = 3 as an example.

Suppose the feature map are labeled A1,A2,…, following a uniform format TP, where T∈{A,B,C,…} represents the channel with values {1,2,3,…,#groups}, $P\in \{1,2,3,\ldots ,\frac{\#channels}{\#groups}\}$ is the feature map in each group. #channels and #groups are the number of channels and groups, respectively.

(1) $$\begin{array}{l} (P-1)\times \#groups+T \end{array}$$

For example:


B1→21

i.e. P=1,T=2


position=(1−1)×3+2=2

That is, before and after channel shuffle, feature map *B* 1’s position was 4 and 2, respectively (see **Figure 1a**).

Therefore, the channel shuffle is a kind of *partial shuffle* rather than *full*. Additionally, we find that the target sizes in chili flower images are generally small, and the importance of different regions varies. This characteristic is especially noticeable in greenhouses due to high-density planting and long shooting distances. Although classic channel shuffle promotes information exchange across channels, it operates globally and ignores differences in distribution within local image regions. This method is suboptimal for accurately identifying small-sized and easily disrupted chili flowers. To improve the model performance for chili flower recognition in complex environments, we propose a localized approach to channel shuffle, which extends 2D channel shuffle to 3D by taking into account interior regions inside feature maps, as shown in **Figure 1**. This method avoids the limitations of partial shuffle, achieving full shuffle.

**Figure 1b** shows the result of shuffling when there are 9 channels, 3 groups, and 3 regions. The positions of feature maps after shuffle are computed by Equation 2. Suppose the labels of feature maps are *A*11*, A*12*,…*, following the uniform format *T P R*, where *T* ∈ {*A, B, C,…*} represents the channel with values {1,2,3,…,#groups}. $P\in \{1,2,3,\ldots ,\frac{\#channels}{\#groups}\}$ denotes the feature map in each group. T and P are the same as in Equation 1. A new parameter R∈{1,2,3,…,#regions} is introduced, where R represents the regions segmented within images. # *channels*, # *groups*, # *regions* denote the number of channels, groups, and regions, respectively.

(2) $$\begin{array}{l} (P-1)\times \#groups+(R-1)\times \#groups\times \#regions+T \end{array}$$

For example:


B12→212

i.e. T=2,P=1,R=2


positon=(1−1)×3+(2−1)×3×3+2=11

That is, the position of B12 is 11 after the shuffle (see **Figure 1b**).

An alternative shuffle calculates the feature map position using Equation 3.

(3) $$\begin{array}{l} (R-1)\times \#regions+(P-1)\times \#groups\times \#regions+T \end{array}$$

Still taking B12 as an example, T=2,P=1,R=2:


position=(2−1)×3+(1−1)×3×3+2=5

Intuitively describing the shuffle mechanism, Equations 2 and 3 respectively represent *vertical shuffle* and *horizontal shuffle* as illustrated in **Figure 1**. Considering the principles behind these two shuffles, we refer to them as *feature map-priority shuffle* and *region-priority shuffle*. For chili flower detection, we empirically select 8 regions based on the size of the feature maps in the dataset.

Compared to classic shuffle, full shuffle not only improves information sharing across channels by minimizing the weakening of inter-channel correlations caused by numerous convolutions, but it also enables the process to better adapt to local characteristics. Furthermore, it provides several advantages in this scenario:

1. Chili flowers are typically small in size and occupy little space in images. Performing shuffle within the region containing the target enhances its distinctiveness in the feature space, thereby improving the representation of small objects.
2. In greenhouses, the background of chili flowers is often highly complex, involving various vegetation, soil, and lighting variations. Traditional shuffles fail to suppress background noise in chili flower representation, compromising accuracy. In contrast, our method effectively reduces background noise, enhancing model robustness.

Additionally, gradients can be incorporated into the model (see **Figure 1b**), which can be referred to as *gradient-enhanced full shuffle* or *edge-enhanced full shuffle*. Natural images typically exhibit rich local structures, which degrade differently during distortion. Local gradients reflect quality to some extent, and analyzing the statistical properties of local gradients can provide valuable insights into noise and quality.

### 3.2 Environment-aware C3k2

YOLO11 detection for chili flowers is significantly hampered in greenhouses by factors including small flower targets, low color contrast, uneven lighting, and severe occlusion. Incorporating environmental data and even time parameters into YOLO11 is important to improving the extraction and reducing the effect of unnecessary redundant data on identification accuracy. C3k2 is one of the key blocks in YOLO11. Through replacing the 1 × 1 conv layer in the C2 module with two consecutive 3 × 3 conv layers, it effectively expands the receptive field with significantly increasing computational complexity. However, when applied to chili flower detection in greenhouse scenarios, C3k2 exhibits two issues:

1. Chili flowers exhibit diversity in size and shape due to different shooting distances and angles. However, C3k2 employs a uniform standard to extract information, resulting in the loss of objects that occupy a small portion of the image during the process.
2. As shown in **Figure 2**, chili flowers are immersed in warm, humid air within greenhouses (see **Figure 2c**). The sunlight pours down through the transparent glass or plastic film. The light remains abundant though somewhat obstructed (see **Figure 2b**). As evening approaches, the light dims. To prevent lodging, rows of chilis are suspended on trellises, with white chili flowers dotting the foliage (see **Figure 2a**). Embedding these complex external cues into the model undoubtedly provides positive guidance for training, but YOLO11 cannot integrate them.

**Figure 2:**
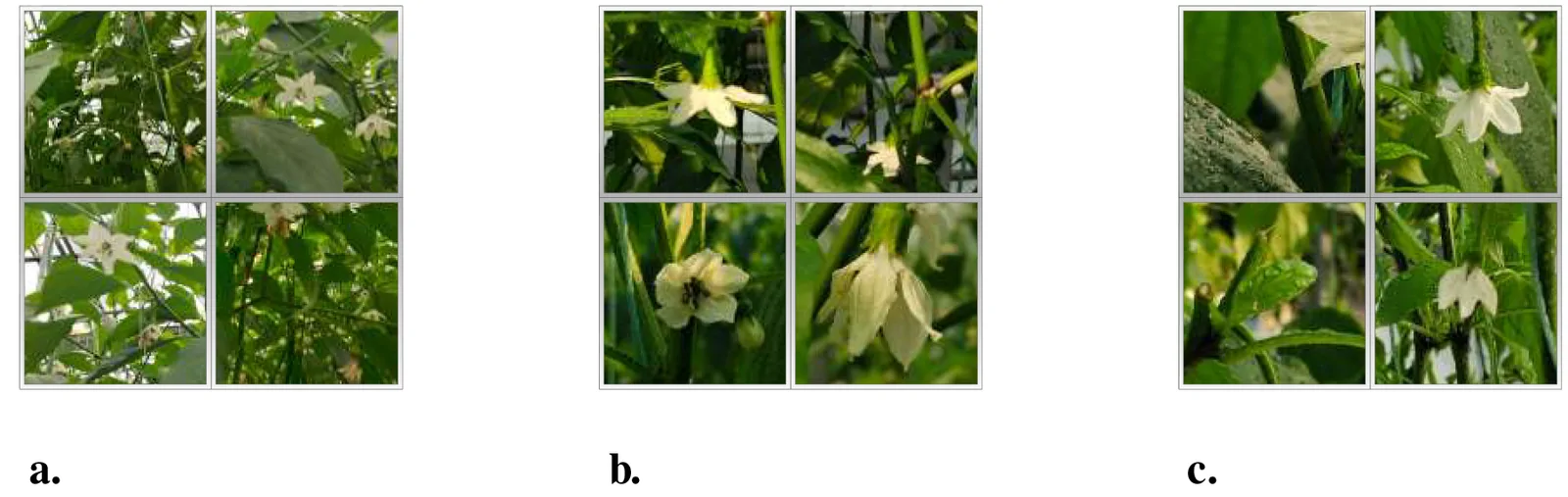
Images obtained from greenhouses. **(a)** Images with high flower density and locally visible. **(b)** images affected by lighting. **(c)** Image of water droplets adhering to the surface of leaves and petals.

In this study, we propose a novel C3k2 module based on external information fusion, which enhances the ability to focus on targets in complex environments by incorporating additional cues. The framework of the improved C3k2 module is shown in **Figure 3**.

**Figure 3:**
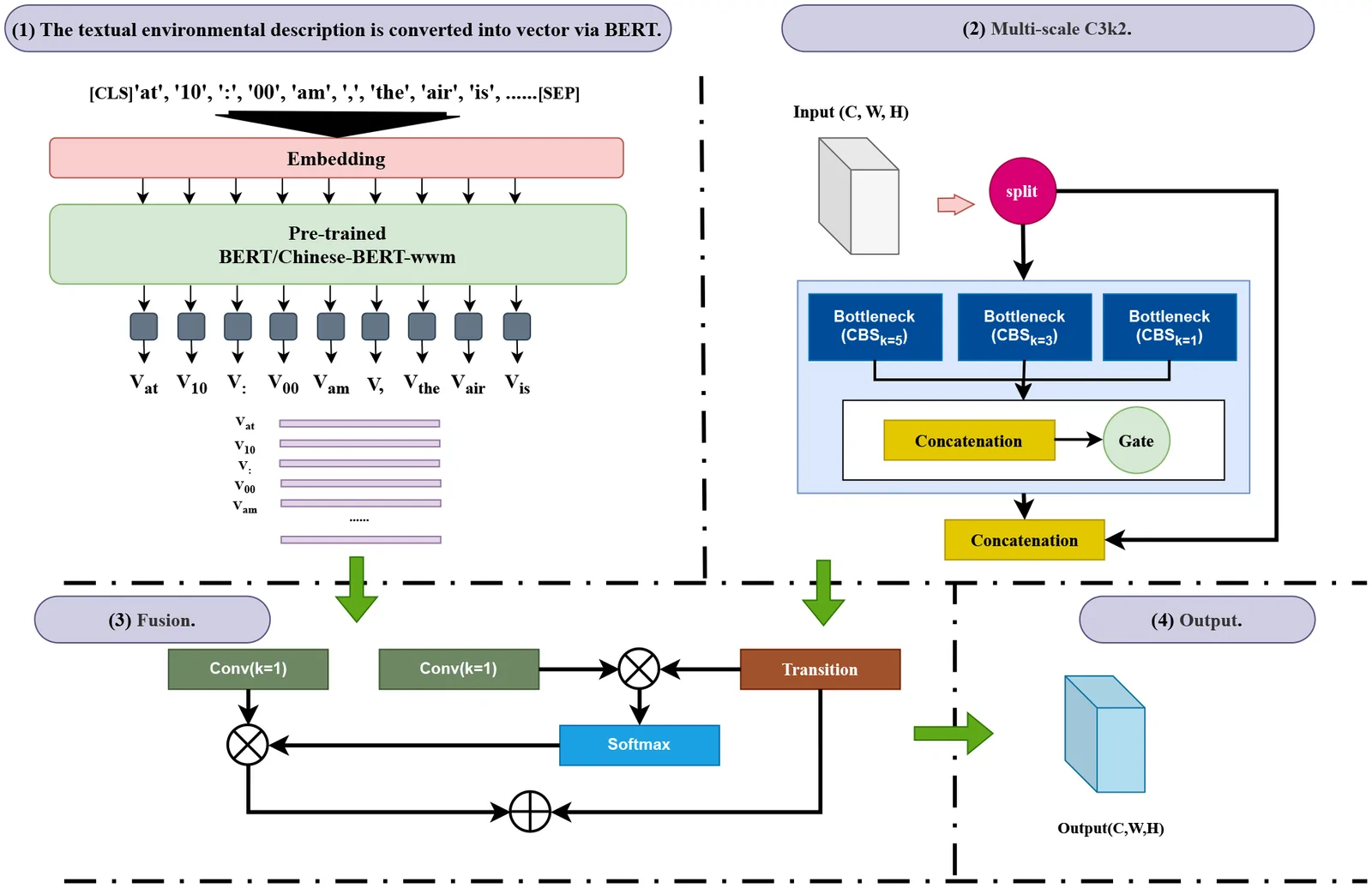
Overall framework of the environment-aware C3k2.

This module runs in two stages:

Stage #1: Unlike the classic C3k2 where the bottleneck employs a constant kernel *k* = 3, we apply multiple kernel (1 × 1, 3 × 3, 5 × 5) to handle chili flowers in diverse perspectives. This enriches gradient information and improves extraction of small objects within images. Multi-scale feature maps are filtered by GATE and concatenated to the other branch after splitting to fit the channels with the external information embedding, yielding *O*_1_ (see Equations 4, 5).

(4) $$\begin{array}{l} i_{1},i_{2}=S(I) \end{array}$$

(5) $$\begin{array}{l} O_{1}=C(G(C(Bo_{k=1}(i_{2}),Bo_{k=3}(i_{2}),Bo_{k=5}(i_{2}))),i_{1}) \end{array}$$

where *S*(·) denotes the split operation, which divides the input *I* into two parts *i*_1_*, i*_2_. *Bo* represents the bottleneck, where three bottlenecks with different kernels, i.e., *k* = 1*, k* = 3*, k* = 5, are employed to achieve multi-scale. *C*(·) stands for concat and *G*(·) is a gate defined by Equation 6.

(6) $$\begin{array}{l} G(x)=conv_{k=1}(x):n\times c\to c \end{array}$$

where c is the channel number of $\frac{I}{2}$, and *n* = 3 here.

Stage #2: To integrate external information into YOLO11, Bert (Devlin et al., 2019) - originally proposed by Google in 2018 — was selected. As a landmark in natural language processing based on the transformer architecture, BERT’s performance and accuracy are remarkable compared to existing models, making it well-suited for this task. There are numerous variants of BERT, such as RoBERTa (Liu et al., 2019), DeBERTa (He et al., 2020), TinyBERT (Jiao et al., 2020), FastBERT (Liu et al., 2020), StructBert (Wang et al., 2019) etc., allowing users to select the appropriate version based on different application scenarios.

**Table 2** shows the number of parameters and suitable application scenarios for six BERT variants. RoBERTa, as an enhanced version of BERT, has a large number of parameters and is slower. DeBERTa, although it has fewer parameters, still requires substantial inference resources. All of these are suitable for applications that prioritize high accuracy. FastBERT and StructBERT each have their unique strengths: FastBERT supports early-exit, which can improve speed to some extent, while StructBERT enables structural analysis. However, both contain around 110 million parameters. Among the various BERT variants, TinyBERT delivers high-speed inference with a small number of parameters, making it the best choice for real-time inference on edge devices. Given the hardware environments, TinyBERT is the best choice.

**Table 2:** Comparative analysis of BERT variants.

Model	Params (M)	Suitability
RoBERTa	125	Server-side high-accuracy tasks
DeBERTa	86	high-accuracy tasks
TinyBERT	14.5(4L)	Edge real-time inference
FastBERT	110	High-throughput server classification
StructBERT	110	Fine-grained semantic matching

We utilize the pre-trained TinyBERT to encode textual environmental information, which is described in natural language.

For example:

Text: “At 10:00 AM, the air is warm and humid,…”
Token: [CLS]’at’, ‘10’, ‘:’, ‘00’, ‘am’, ‘,’, ‘the’, ‘air’, ‘is’, ‘warm’, ‘and’, ‘humid’,…[SEP]

The parameters feed are fed into the BERT to generate the encoded environmental vector *O*_2_(*T, D*), where T is the natural language described input text. D denotes the dimension of the word vector set to 100 by default. The fusion of *O*_1_ and *O*_2_ generates the final output (see Equations 7, 8).

(7) $$\begin{array}{l} z_{1}=Softmax(Conv_{k=1}(O_{2}(T,D))⊗T(O_{1})) \end{array}$$

(8) $$\begin{array}{l} Output=(Conv_{k=1}(O_{2}(T,D))⊗z_{1})⊕T(O_{1}) \end{array}$$

where *T*(·) represents the transition to align the dimensions of both parties in the calculation. ⊗ and ⊕ denote matrix multiplication and addition, respectively.

Textual descriptions are manually constructed in our setting based on the known features of the greenhouse environment where data were gathered, ensuring that the method is scalable. They are encoded by BERT to guide the training process but not create a runtime dependency, ensuring reproducibility. Deployment requires no additional manual intervention. Redefining textual descriptions for different environments is a straightforward one-time configuration.

### 3.3 P-rectified semi-inner powerful IoU

Throughout the development of object detection, Intersection over Union (IoU) has consistently served as the primary metric for evaluating the alignment between predicted and ground-truth bounding boxes. It is defined by Equation 9.

(9) $$IoU=\frac{Intersection}{Union}=\frac{B_{P}\cap B_{G}}{B_{P}\cup B_{G}}$$

where $B_{g}$ and $B_{g}$ are the predicted and ground-truth bounding boxes, respectively. Since IoU was initially applied to object detection scenarios, it has undergone many improvements: GIoU (Rezatofighi et al., 2019), DIoU (Zheng et al., 2020), CIoU, SIoU (Gevorgyan, 2022), WIoU (Tong et al., 2023), alpha IoU (He et al., 2021), Focal S/G/D/E/CIoU (Zhang et al., 2019), and Inner-IoU (Zhang et al., 2023a), Focaler-IoU (Zhang and Zhang, 2024), Powerful IoU (Liu et al., 2024), MPDIoU (Siliang and Yong, 2023). These improvements have evolved from a single overlap metric into complex loss functions that take into consideration various geometric relationships—such as position and shape—thereby enhancing both accuracy and convergence speed. However, the variety of the real world is not well addressed by the current IoU, especially in complex greenhouses. By focusing on the interior of bounding boxes, Inner-IoU reduces interference from external backgrounds, thereby improving detection speed. This characteristic is particularly important for detecting chili flowers in greenhouses. However, Inner-IoU does not account for factors such as shape, position, and relative size. Powerful IoU (PIoU), defined by Equations 10, 11.

(10) $$p=(\frac{dw_{1}}{w_{gt}}+\frac{dw_{2}}{w_{gt}}+\frac{dh_{1}}{h_{gt}}+\frac{dh_{2}}{h_{gt}})/4$$

(11) $$\begin{array}{l} PIoU=1-IoU+1-e^{-p^{2}} \end{array}$$

where *dw*_1_, *dw*_2_, *dh*_1_, *dh*_2_ denote the distance between the four edges of the predicted box and the GT box. *w_gt_*, *h_gt_* are the width and height of the GT box. Minimizing PIoU guides the predicted box to move toward the GT box along an approximate straight-line path. PIoU incorporates object size as an adaptive penalty factor *p*, effectively mitigating slow convergence caused by unnecessary expansion of predicted boxes during training. Building upon the design principles of Inner-IoU and Powerful IoU, Inner Powerful IoU is proposed. Its concept involves calculating inner bounding boxes within both predicted and ground truth (GT) boxes, with IoU computation entirely based on inner boxes. This approach accelerates model convergence, reduces external interference, and accounts for factors like shape and position. However, computing two inner boxes increases the computational complexity of IoU. In this study, we propose Semi-inner Powerful IoU (see **Figure 4**), which accelerates model convergence and enhances chili flower recognition performance by more flexible ratio settings.

**Figure 4:**
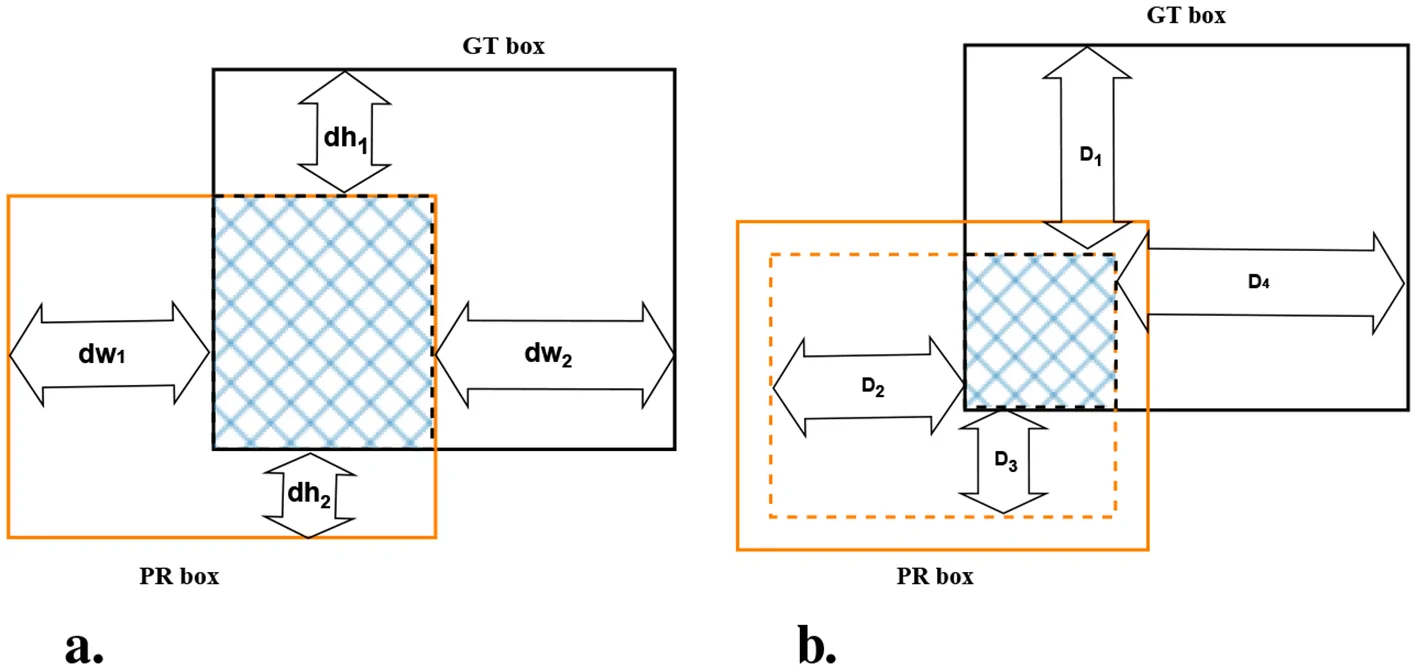
IoU. **(a)** Powerful-IoU **(b)** Semi-inner Powerful IoU.

Unlike the two inner boxes proposed by Inner-IoU, Semi-inner Powerful IoU only requires one inner box within the predicted box. Since the GT box is manually annotated, it can theoretically precisely delineate the object boundary. Its vertices are $\left(x_{1}^{I},y_{1}^{I}\right)$ (top-left) and $\left(x_{2}^{I},y_{2}^{I}\right)$ (bottom-right). The GT box’s vertices are $\left(x_{1}^{G},y_{1}^{G}\right)$ (top-left) and $\left(x_{2}^{G},y_{2}^{G}\right)$ (bottom-right). The predicted box’s vertices are $\left(x_{1}^{P},y_{1}^{P}\right)$ (top-left) and $\left(x_{2}^{P},y_{2}^{P}\right)$ (bottom-right) (see Equations 12–15).

(12) $$\begin{array}{l} x_{1}^{I}=x_{c}^{P}-\frac{W_{P}}{2}\times r \end{array}$$

(13) $$\begin{array}{l} y_{1}^{I}=y_{c}^{P}-\frac{H_{P}}{2}\times r \end{array}$$

(14) $$\begin{array}{l} x_{2}^{I}=x_{c}^{P}+\frac{W_{P}}{2}\times r \end{array}$$

(15) $$\begin{array}{l} y_{2}^{I}=y_{c}^{P}+\frac{H_{P}}{2}\times r \end{array}$$

where $\left(x_{c}^{P},y_{c}^{P}\right)$ are the center point of the predicted box, which are also the center point of the inner box. *W_P_*, *H_P_* are the width and height of the predicted box, respectively. *r <* 1 is the scaling ratio for the inner box.

The intersection over union for Semi Inner-IoU is modified as Equations 16–18.

(16) $$IoU_{semi}=\frac{B_{G}\cap B_{I}}{B_{G}\cup B_{I}}$$

(17) $$\begin{array}{ll} B_{G}\cap B_{I} & =\max\;(\min\;(y_{2}^{G},y_{2}^{I})-\max\;(Y_{1}^{G},Y_{1}^{I}),0)\\ \times \max\;(\min\;(x_{2}^{G},x_{2}^{I})-\max\;(x_{1}^{G},x_{1}^{I}),0) \end{array}$$

(18) $$\begin{array}{ll} B_{G}\cup B_{I} & =(x_{2}^{G}-x_{1}^{G})\times (y_{2}^{G}-y_{1}^{G})\\ +(x_{2}^{I}-x_{1}^{I})\times (y_{2}^{I}-y_{1}^{I})-B_{G}\cap B_{I} \end{array}$$

where $B_{G}\;$ represents the GT box, where $B_{I}$ is the inner box. The formula for the target size-adaptive penalty factor p and loss function in Powerful IoU is as Equations 19, 20.

(19) $$p=\frac{1}{4}(\frac{D_{2}+D_{4}}{W_{G}}+\frac{D_{1}+D_{3}}{H_{G}})$$

(20) $$L=1-IoU+(1-e^{-p^{2}})$$

The definition of distances *D*_1_*, D*_2_*, D*_3_*, D*_4_ (see Equations 21–24) is no longer between the predicted box and the GT box but between the inner box and the GT box:

(21) $$D_{1}=|\min\;(y_{1}^{G},y_{2}^{G})-\min\;(y_{1}^{I},y_{2}^{I})|$$

(22) $$D_{2}=|\min\;(x_{1}^{G},x_{2}^{G})-\min\;(x_{1}^{I},x_{2}^{I})|$$

(23) $$D_{3}=|\max\;(y_{1}^{G},y_{2}^{G})-\max\;(y_{1}^{I},y_{2}^{I})|$$

(24) $$D_{4}=|\max\;(x_{1}^{G},x_{2}^{G})-\max\;(x_{1}^{I},x_{2}^{I})|$$

However, after introducing the inner box, $D_{4}$ increases by $\frac{1-r}{2}W_{P}$, $D_{2}$ decreases by $\frac{1-r}{2}W_{P}$, then their sum remains unchanged. Similarly, $D_{1}$ increases by $\frac{1-r}{2}H_{P}$ while $D_{3}$ decreases by $\frac{1-r}{2}H_{P}$, so their sum also remains unchanged. Therefore, the inner box does not affect the p. However, since the convergence process takes the inner box as the reference, this leads to the phenomenon where the predicted box becomes larger than the GT box (as shown in **Figure 5** below).

**Figure 5:**
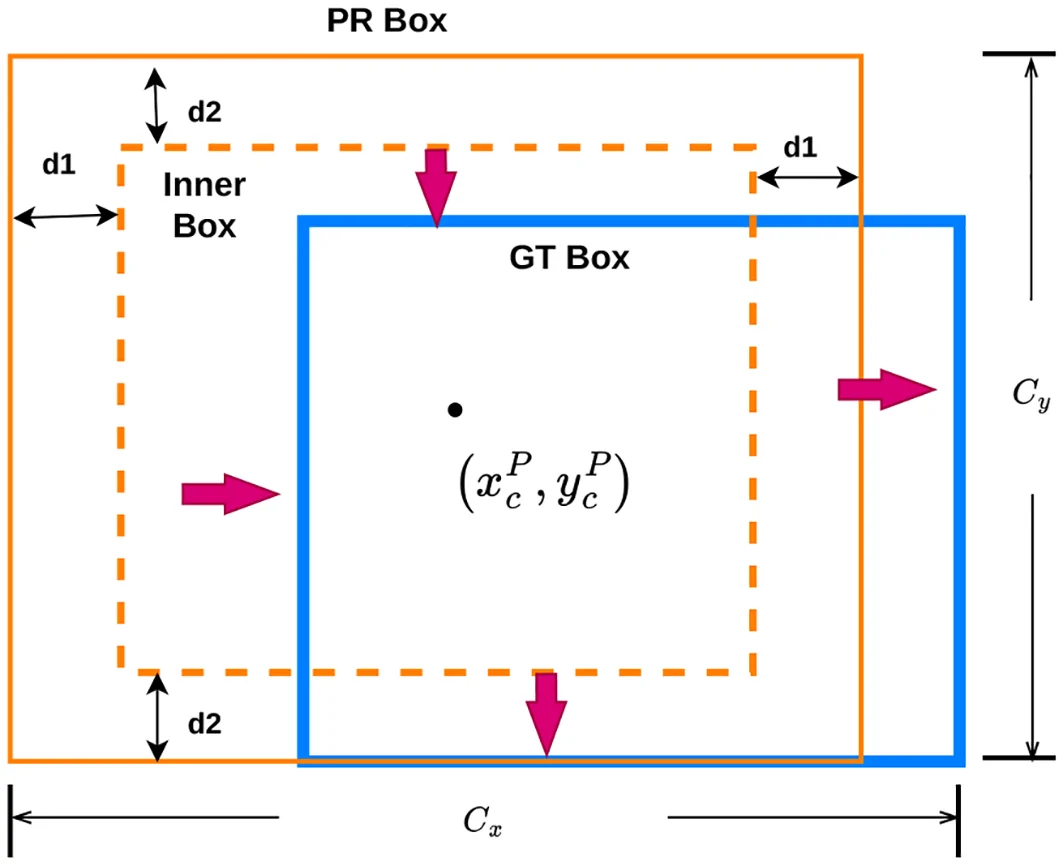
The reason for the phenomenon that the predicted box is larger than the GT box. $\left(x_{c}^{P},y_{c}^{P}\right)$ is the center point of the predicted box. $d1=\frac{W_{G}-W_{P}}{2}$ denotes the width difference between the predicted and inner boxes, $d2=\frac{H_{G}-H_{P}}{2}$ represents the height difference between the predicted and inner boxes.

In order to make the predicted box more accurately approximate the GT box, the p-rectified semi-inner powerful IoU is proposed, which further accounts for the size discrepancy between the predicted and GT boxes. The TYPE-I expression for the p-factor is given by Equations 25–27.

(25) $$p^{TYPE-I}=\frac{1}{4}(\frac{D_{2}+D_{4}}{W_{G}}+\frac{D_{1}+D_{3}}{H_{G}})+\gamma (\frac{\left|W_{G}-W_{P}\right|}{C_{x}}+\frac{\left|H_{G}-H_{P}\right|}{C_{y}})$$

(26) $$C_{x}=|\max\;(x_{2}^{G},x_{2}^{P})-\min\;(x_{1}^{G},x_{1}^{P})|$$

(27) $$C_{y}=|\max\;(y_{2}^{G},y_{2}^{P})-\min\;(y_{1}^{G},y_{1}^{P})|$$

where *γ* serves as a trade-off factor, namely *rectification factor*, that determines how closely the predicted box aligns with the GT box. Therefore, the hyperparameter *γ* requires fine-tuning based on recognition performance in real-world scenarios. *W_G_, H_G_* are the width and height of the GT box. *W_P_, H_P_* are the width and height of the predicted box. Regarding *C_x_* and *C_y_*, the inner box can also serve as a reference, with Equations 28, 29.

(28) $$C_{x}^{I}=|\max\;(x_{2}^{G},x_{2}^{I})-\min\;(x_{1}^{G},x_{1}^{I})|$$

(29) $$C_{y}^{I}=|\max\;(y_{2}^{G},y_{2}^{I})-\min\;(y_{1}^{G},y_{1}^{I})|$$

where $\left(x_{1}^{I},y_{1}^{I}\right)$, $\left(x_{2}^{I},y_{2}^{I}\right)$ are the top-left and bottom-right vertices of the inner box. $\left(x_{1}^{G},y_{1}^{G}\right)$, $\left(x_{2}^{G},y_{2}^{G}\right)$ represent the top-left and bottom-right vertices of the GT box. The corresponding TYPE-II expression for the p-factor is as given by Equation 30.

(30) $$p^{TYPE-II}=\frac{1}{4}(\frac{D_{2}+D_{4}}{W_{G}}+\frac{D_{1}+D_{3}}{H_{G}})+\gamma (\frac{\left|W_{G}-W_{P}\right|}{C_{x}^{I}}+\frac{\left|H_{G}-H_{P}\right|}{C_{y}^{I}})$$

Comparison: The rectified term of $p^{TYPE-I}$ exhibits low correlation with the inner box, being determined solely by the GT and predicted boxes. So, it shows the advantage of converging stably in applications, though its convergence speed is slower than that of $p^{TYPE-II}$. The rectified term of $p^{TYPE-II}$ is closely related to the inner box, with its convergence speed influenced by the scaling ratio *r*:

As shown in **Figure 6**, when r increases, the rectified term $\frac{\left|W_{G}-W_{P}\right|}{C_{x}^{I}}+\frac{\left|H_{G}-H_{P}\right|}{C_{y}^{I}}$ increases, thereby amplifying its effect on the loss function. Conversely, when r decreases, the rectified term diminishes, thereby weakening its influence on the loss function. That is, when r↗, then $C_{x}^{I}↘$, $C_{y}^{I}↘$, so $(\frac{\left|W_{G}-W_{P}\right|}{C_{x}^{I}}+\frac{\left|H_{G}-H_{P}\right|}{C_{y}^{I}})↗$. On the contrary, when r↘, then $C_{x}^{I}↗$, $C_{y}^{I}↗$, so $(\frac{\left|W_{G}-W_{P}\right|}{C_{x}^{I}}+\frac{\left|H_{G}-H_{P}\right|}{C_{y}^{I}})↘$. Therefore, the loss rises with increasing *r* and falls with decreasing *r*.

**Figure 6:**
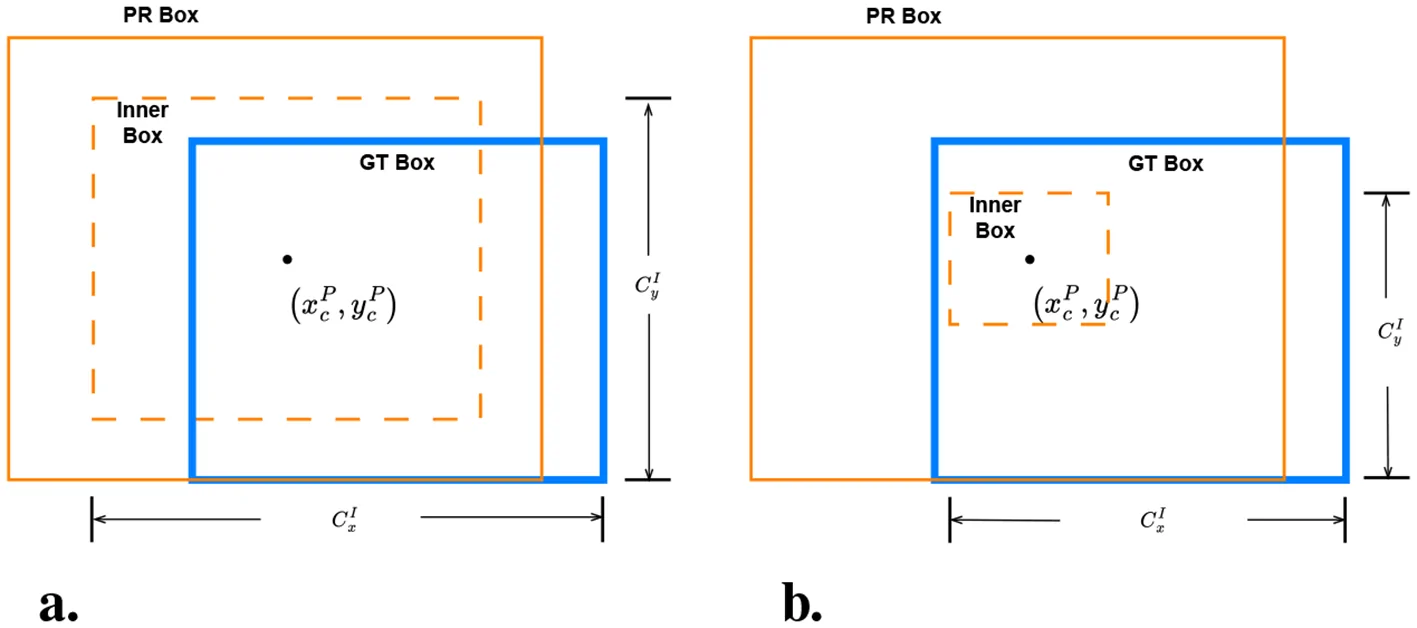
When the predicted box and GT box remain fixed in position, the relative positions of the inner box and GT box change with different *r*, resulting in differences between $C_{x}^{I}$ and $C_{y}^{I}$. **(a)** Big *r*. **(b)** Small *r*.

If the loss function continues to be computed using the original method, box loss may become unstable and fail to converge during training. Since this result is counterintuitive, the loss function expression is modified see Equation 31.

(31) $$\begin{array}{l} L_{v1}^{new}=IoU_{semi}+1-e^{-p} \end{array}$$

To focus more on medium-quality anchor boxes, Powerful IoU introduces a non-monotonic attention layer, thereby forming the loss function of version 2. The formula *L _v_*_2_ is defined as: *L_v_*_2_ = 3*m*(*λq*) · *L _v_*_1_, where *m*(*λq*) (see Equations 32, 33) denotes the non-monotonic attention layer. The formula *m*(*x*) is as follows:

(32) $$\begin{array}{l} m(x)=3x\cdot e^{-x^{2}} \end{array}$$

$$∵q=e^{-p}$$

(33) $$\begin{array}{l} ∴m(\lambda q)=3\lambda e^{-p}\cdot e^{-{\left(\lambda e^{-p}\right)}^{2}} \end{array}$$

This non-monotonic attention layer *m*(*λq*) was proven as effective during training, yet it defies intuition to some extent, involves high computational complexity, and lacks a well-established theoretical foundation, posing challenges for practical applications.

Inspired by the sigmoid function, as follows:


$$S(x)=\frac{1}{1+e^{-x}}\Rightarrow S^{\prime }(x)=\frac{e^{-x}}{{\left(1+e^{-x}\right)}^{2}}$$

where $S^{\prime }(x)$ stands for the derivative of the sigmoid. We designed a novel non-monotonic attention layer featuring a more concise and intuitive form, higher computational efficiency, and equal optimization performance. The formula is given by Equations 34, 35.

(34) $$\begin{array}{l} m(p)=\frac{e^{-p}}{{\left(1+e^{-\lambda p}\right)}^{2}} \end{array}$$

(35) $$\begin{array}{l} L_{v2}^{new}=3m(p)\times L_{v1} \end{array}$$

where *λ* serves as a hyperparameter to tune the function for optimal results. The monotonicity of *m*(*p*) is similar to that of *m*(*λq*), with its maximum value also occurring within the range [0, 1], enabling greater focus on medium-quality anchor boxes. The curves of *m*(*λq*) and *m*(*p*) are shown in **Figure 7**, which intuitively reflects their similarity. We conclude from experiments that in practical situations, meticulously tweaking the *λ* of *m*(*p*) can infinitely approximate *m*(*λq*), thereby achieving optimal convergence during training.

**Figure 7:**
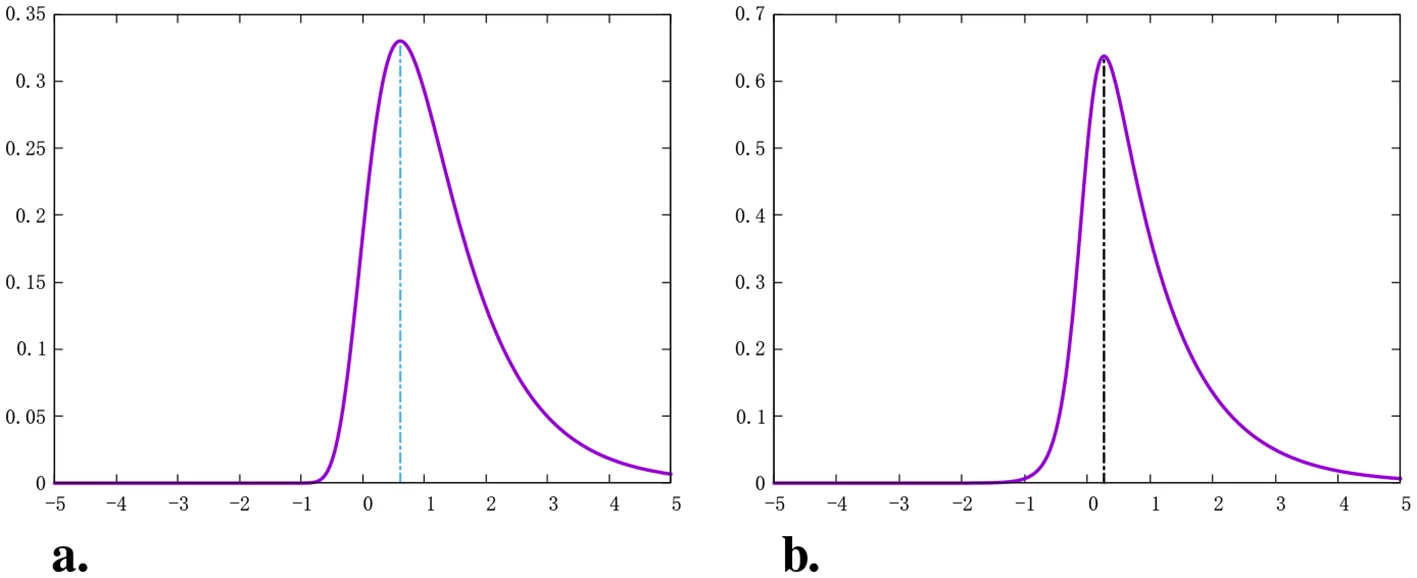
Comparison of two function curves. **(a)** *m*(*λq*)*, λ* = 1.3. **(b)** *m*(*p*)*, λ* = 3.

Compared to other IoUs, Powerful-IoU considers multiple factors such as box shape, position, and relative size. It effectively suppresses overfitting during regression, enhances convergence speed and stability, and performs reliably in complex scenarios like greenhouses. Semi-inner Powerful IoU builds upon the classic.

Powerful IoU and Inner-IoU while gaining prominence through its more flexible hyperparameter *r* and reduced complexity (eliminating one inner box). Semi-inner Powerful IoU can also incorporate exponential deformation, generalizing it into exponential IoU loss, namely *α* Semi-inner Powerful IoU, given by Equation 36.

(36) $$\begin{array}{l} L=IoU_{semi}^{\alpha }+{\left(1-e^{-p}\right)}^{\alpha } \end{array}$$

Bounding box regression accuracy is increased by choosing a suitable *α*. Furthermore, the novel *α*-version localization loss demonstrates improved resistance to limited datasets and noisy boxes.

### 3.4 Improved YOLO11 framework

One of YOLO’s main advantages is its inference speed, which YOLO11 retains while striking a balance between speed and accuracy. YOLO11 removes the bottleneck of non-maximum suppression (NMS), simplifies the operation flow and parameter tuning, making deployment more convenient. Its lightweight design makes YOLO11 well-suited for embedded environments with limited memory. However, it still faces performance limitations in greenhouses. Particularly under drastic light fluctuations and leaf occlusion, YOLO11 may exhibit detection errors or omissions dramatically. To address these issues, our method incorporates improvements in three aspects: full shuffle, conditional multi-scale C3k2, and an improved loss function. The refined YOLO11 framework is illustrated in **Figure 8**.

**Figure 8:**
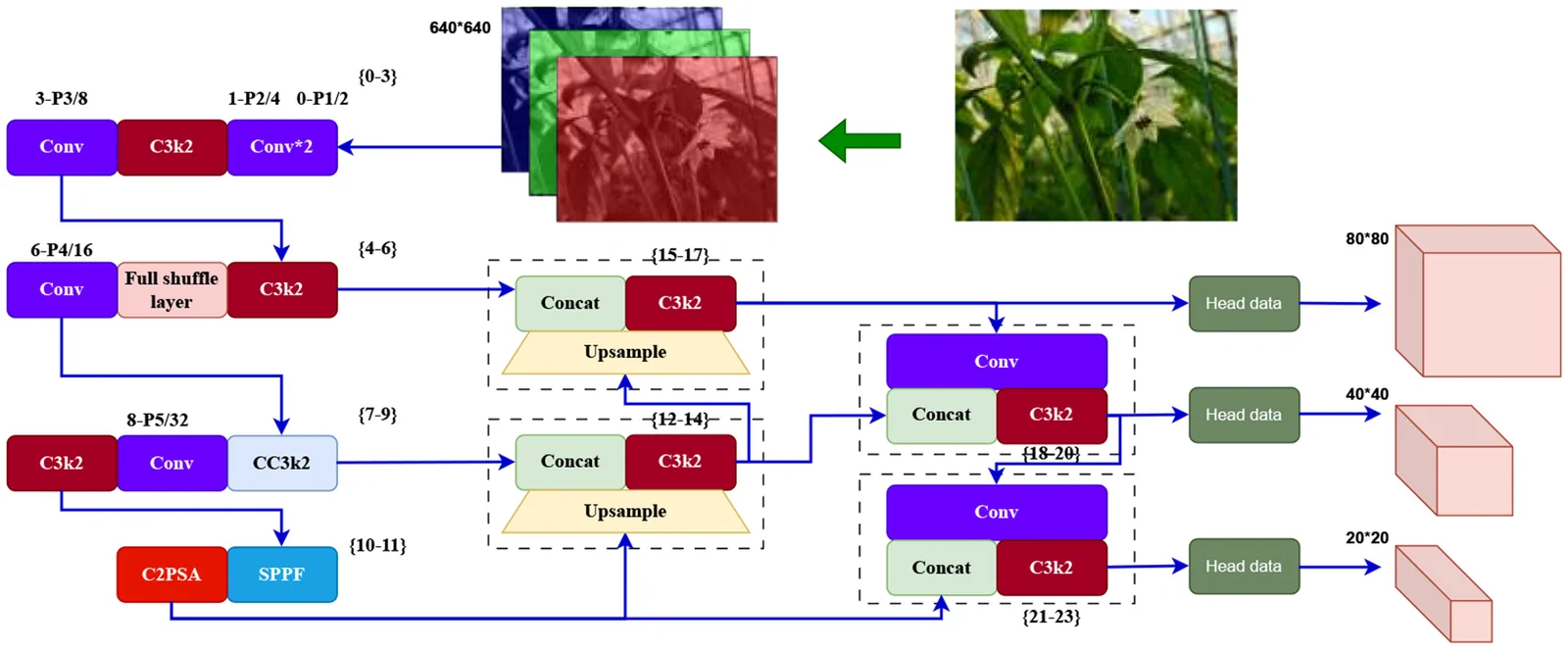
The framework of improved YOLO11.

## 4 Experimental results

### 4.1 Experimental environment and parameter configuration

All experiments in this study were conducted on a unified platform with consistent hardware and software setups to ensure a fair evaluation of chili flower detection performance. This method guarantees reliable and repeatable findings by eliminating the influence of external factors, such as hardware conditions and software versions, on experimental results, training efficiency, and accuracy.

The hardware environment for this experiment comprised high-performance computing devices and graphics processing units (GPUs). The specific configuration is as follows:

- CPU: Intel(R) Xeon(R) Silver 4210
- GPU: NVIDIA Tesla T4 16GB
- RAM: 128G DDR4
- External storage: 2TB SSD

This experiment employs mainstream deep learning frameworks and installs the necessary dependency libraries:

- OS: Windows Server 2016 Standard
- Deep learning framework: Pytorch 2.2.2+cu118
- CUDA: CUDA 11.8

### 4.2 Data preparation

We employed two datasets in our experiments: coco128 and our self-built chili flower dataset. The coco dataset was primarily used for model pre-training and evaluating certain experimental results on public datasets. To portray the imbalance of objects in practical scenarios, the self-built dataset sought to capture the natural characteristics of flowers in real greenhouses. Images were collected at the shouguang facility vegetable high-tech demonstration park, shandong province, china. To guarantee quality and representativeness, the data gathering was strictly controlled. Key parameters for data acquisition are summarized in **Table 3**.

**Table 3:** Key parameters for data acquisition.

Key parameters	Description
Location	No. 9 Hall of Shouguang Vegetable High-Tech Demonstration Park (Weifang University of Science and Technology Exhibition Hall) Shandong Province, China
Latitude and Longitude	Longitude: 118.82007 Latitude: 36.85554
Data collection equipment	Device: Vivo S16e Model number: V2239A Hardware version NO. MP 0.1 Processor:2.8GHz; E1080 Octa-core
Image resolution	4080-3060
Time interval per day	8:00-11:30, 14:00-17:00
Distance	0.01-1.5 meter
Environmental conditions	Various related regional environmental conditions descriptions. Such as sunny, cloudy, temperature, relative humidity, and occlusion.
Total number of images	1156

The process fully accounts for various environmental conditions, such as diverse perspectives, lighting fluctuations, scene obstructions, and backgrounds like soil, hemp rope, and brackets. This diversity is crucial to improving the generalization ability of the model and its applicability in the real world. Rich metadata, such as precise geolocation, timestamps, and ambient temperature and humidity, is included with each image, offering important contextual data for model training. Representative samples from the dataset under various conditions are shown in **Figure 9**.

**Figure 9:**
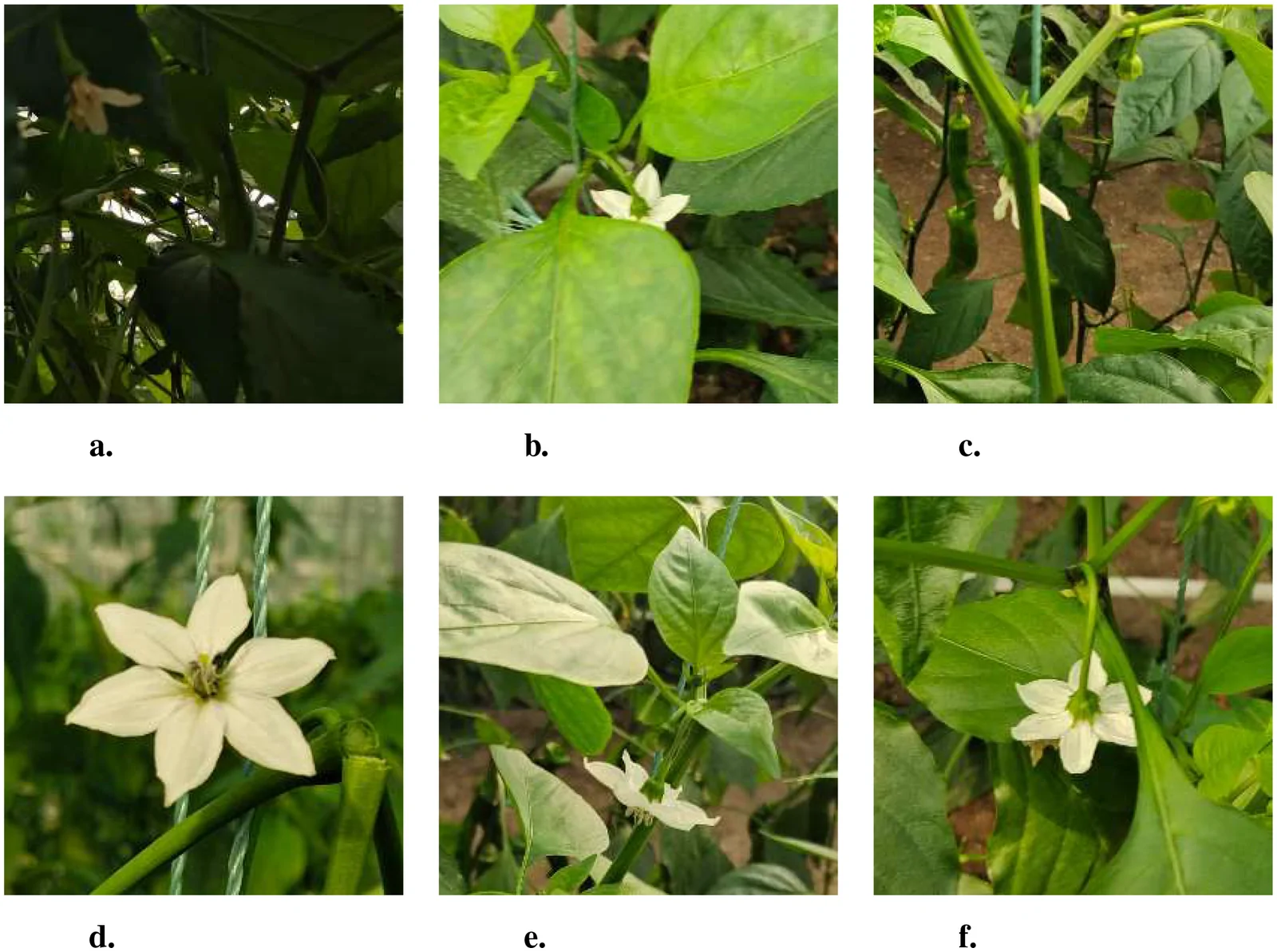
Shows six samples of chili flowers with various states in the dataset: **(a)** Dim light **(b)** Slightly obstructed **(c)** Severely obstructed **(d)** Front view **(e)** Side view **(f)** Back view.

Our dataset realistically represents the natural distribution of chili flowers under different environmental conditions with imbalance issues obviously. **Table 4** shows the sample counts and corresponding proportions under various conditions.

**Table 4:** #Sample and proportion for different conditions of our dataset.

Category	#Sample	Percentage
Bright light	124	10.7%
Dim light	167	14.4%
Slightly obstructed	178	15.4%
Severely obstructed	63	5.4%
Front view	136	11.8%
Side view	253	21.9%
Back view	235	20.3%
Total	1156	100%

The dataset contains the fewest samples with severely obstructed, as this issue was effectively mitigated during data collection by adjusting shooting angles. In contrast, slightly obstructed frequently occurred among densely planted chilies in greenhouses. Since our data collection primarily took place during the daytime, sunlight in greenhouses was filtered by the structure, resulting in a lower probability of bright light compared to dim light. Additionally, chili flowers typically bloom downward, making it easier to capture their sides and backs compared to their fronts. As a result, front-view samples are relatively scarce in the dataset.

The imbalance reflects the relative frequency of chili flowers in greenhouses, which presents us with real-world challenges. The detection of rare state flowers in real applications highlights the importance and difficulty, especially when the shooting distance is far, and the sample size of the side and occlusion of chili flowers is much smaller than that of other categories.

To address the issue of imbalance, we first employed random sampling to ensure uniform distribution of samples across each category in the training, validation, and test sets, with 8:1:1. Subsequently, we applied multiple data augmentations to the training set, including rotation, translation, cropping, flipping, and brightness adjustment.

Gamma correction is primarily used to modify the RGB space. It selectively alters the contrast in low and high-gray-scale regions by varying the *γ* value. The formula is as Equation 37.

(37) $$\begin{array}{l} I(x)={\left(x+\xi \right)}^{\gamma } \end{array}$$

where *x* and *I* (*x*) represent the input and output images, respectively, *ξ* denotes the compensation coefficient, and *γ* is the Gamma coefficient. *γ* is an important parameter in the process. *γ >* 1 increases high-gray-scale regions of the image, whereas *γ <* 1 impacts low-gray-scale regions.

**Figure 10** illustrates the effects of five preprocessing techniques. Through preprocessing, sparse samples are augmented, ensuring overall dataset diversity. This method helps balance performance, particularly improving detection ability in rare states.

**Figure 10:**
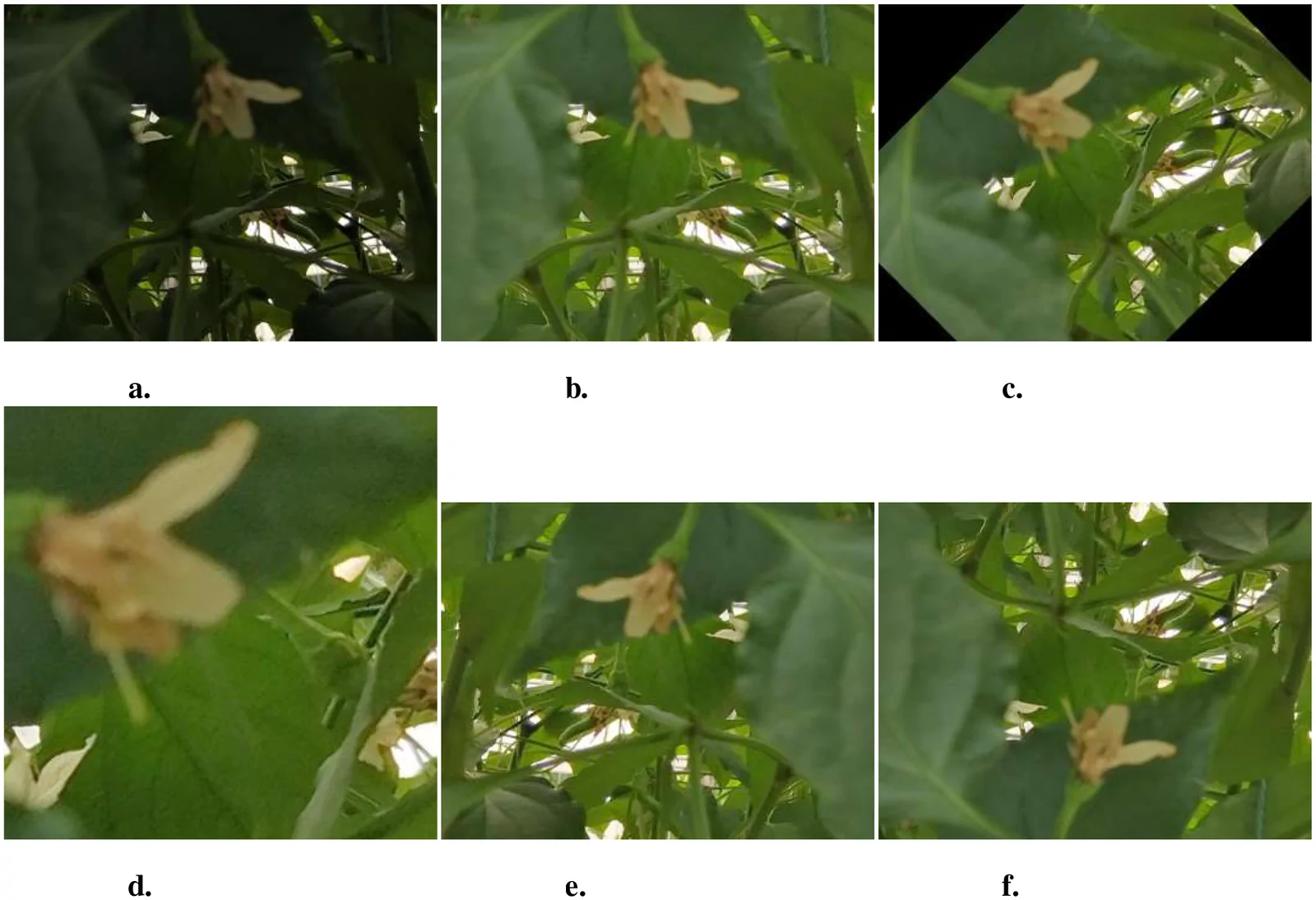
Samples after preprocessing. **(a)** Original image **(b)** Brightness adjustment by Gamma correction *γ* = 0.5 **(c)** Rotation **(d)** Cropping **(e)** Flipping Left→ Right **(f)** Flipping Top→ Bottom.

After data augmentation, the total number of samples in the original dataset was expanded to 1,656—about 1.5 times its original size. Notably, for small classes such as severely obstructed and bright light, the samples increased from 63 and 124 to 203 and 207, respectively. The data augmentation effectively mitigated the imbalance, improved the model’s recognition capability for small classes, and provided a robust foundation for enhancing the model’s generalization ability.

### 4.3 Model training

This study builds upon YOLO11n with enhancements. Loading pre-trained YOLO11n weights from the MS COCO dataset is a reasonable option for accelerating convergence, as it leverages prior knowledge in extracting general features. Input images are sized at 640 × 640. To mitigate initial oscillation, *learning_rate* = 0.001 is employed alongside a warm-up strategy. The warm-up is set to 5 epochs, with cosine annealing applied to gradually decay the learning rate, ensuring smooth convergence toward the optimum. Training is conducted for 1000 epochs. Within the limits of GPU memory, batch size is set to 4 to stabilize gradient descent. At the same time, the Adam optimizer is selected. To prevent overfitting, given the small batch size (4), we adopt an early stopping strategy with a patience of 50 epochs based on the validation loss. We also employ 10% of the dataset as a validation set and conduct repeated experiments to ensure result stability.

We recorded the changes in the model’s loss function and accuracy during training, as shown in **Figure 11**. **Figure 11a** shows that the loss function fluctuated and increased slightly in the early stages due to warm-up. However, it began to decline about the 20th epoch. Although the speed of decrease slowed by the 1000th epoch, the overall downward trend continued. Training accuracy remained on an upward track, peaking at over 80% as shown in **Figure 11b**, with no signs of deceleration.

**Figure 11:**
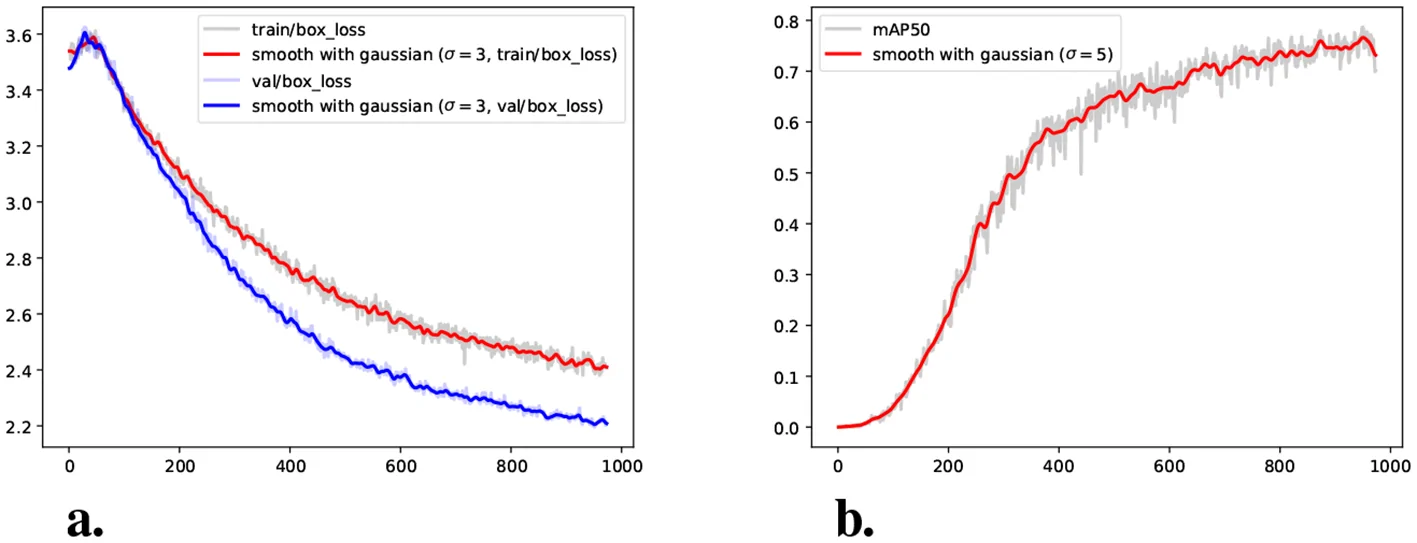
Loss values and evaluation metrics change curves during training YOLO11.

### 4.4 Comparative study

To better evaluate the performance of our method, this experiment selected several mainstream models — such as YOLOv8, YOLO12, Faster R-CNN, RT-DETR, and baseline (YOLO11) - and ran them on our self-built chili flower dataset and coco dataset. These models were trained on the same hardware environment. **Tables 5**, **6** summarizes and compares the experimental results.

**Table 5:** Comparison of results on chili flower dataset.

Methods	P(%)	R(%)	Params(M)	Latency(ms/img)	FPS	△Latency (vs baseline)	GFLOPS	mAP50(%)	△(vs baseline)	mAP50-95(%)
YOLOv8	71.4	63.2	3.2	9.5	105	+1.2	8.9	78.4	+1.1	47.4
Baseline	72.0	65.7	2.6	8.3	120	–	6.5	77.3	–	48.1
YOLO12	69.2	72.5	2.6	11.4	88	+3.1	6.3	81.3	4.0	46.1
RTDETR	78.5	76.0	32.9	55.6	18	+47.3	103.5	81.2	+3.9	45.6
Faster R-CNN(ResNet50-FPN)	66.5	55.3	48.3	21.3	47	+13.0	216.2	68.3	-9.0	42.7
Our method	81.1	75.6	3.2	8.6	117	+0.3	9.3	84.8	+7.5	50.2

**Table 6:** Comparison of results on coco dataset.

Methods	P(%)	R(%)	Latency (ms/img)	FPS	△Latency (vs baseline)	mAP50(%)	△(vs baseline)	mAP50-95(%)
YOLOv8	65.2	52.7	5.3	190	+0.2	61.6	-6.3	45.3
Baseline	68.6	60.6	5.1	195	–	67.9	–	50.4
YOLO12	66.1	56.5	8.3	120	3.2	63.1	-4.8	40.6
RTDETR	72.4	65.2	43.5	23	+38.4	70.7	+2.8	48.5
Faster R-CNN(ResNet50-FPN)	69.3	58.0	16.9	59	+11.8	54.2	-13.7	35.1
Our method	74.8	69.6	5.8	173	+0.7	71.8	+3.9	51.5

Batch size=8.

The mAP shows significant improvement on the chili flower dataset. YOLOv8 and YOLO11 had mAP50 scores of 78.4 and 77.3, respectively, when detecting chili flowers in the complex greenhouse environment. With mAP50 = 84.8, our method outperformed YOLOv8 and YOLO11 by 6.4 and 7.5, respectively, indicating a substantial improvement.

To improve training speed, batch size = 8 was selected for the coco because the images in this dataset are generally small in size. Experimental results show that mAP50 and mAP50–95 improved by 3.9% and 1.1%, respectively, over the baseline. The FPS was 173, indicating a slight decline compared to the baseline. Although coco is not an agricultural dataset, this experiment demonstrates the method’s generalizability, providing greater confidence for real-world deployment.

The experimental results show that our method achieves a satisfactory balance among accuracy, stability, speed, and model complexity, confirming its practical value for smart greenhouse applications. In summary, our method illustrates stable and competitive performance for chili flower detection within complex greenhouse environments.

### 4.5 Ablation experiment

This study proposes three enhancements to improve YOLO11’s accuracy in detecting chili flowers within complex greenhouse backgrounds: full shuffle, environment-aware C3k2, and P-rectified Semi-inner Powerful IoU. The experimental design aims to validate the impact of each enhancement on YOLO11, thereby demonstrating its advantages.

**Table 7** shows each proposed module on the number of parameters, mAP50, and mAP50-95. The experimental results reveal the following:

**Table 7:** Results of ablation experiments. Full shuffle (B01), environment-aware C3k2 (B02), and Prectified semi-inner powerful IoU (B03).

Methods	P(%)	R(%)	Params(M)	Latency (ms/img)	FPS	△Latency (vs baseline)	mAP50(%)	△(vs baseline)	mAP50-95(%)
Baseline	72.0	65.7	2.6	8.3	120	–	77.3	–	48.1
+B01	73.4	68.1	3.0	8.8	114	+0.5	80.8	+3.5	47.6
+B02	70.6	68.3	2.8	9.4	106	+1.1	77.2	-0.1	46.7
+B03	75.9	71.1	2.6	7.5	134	-0.8	79.3	+2.0	49.2
+B01, B02	76.8	71.7	3.2	9.2	109	+0.9	82.7	+5.4	49.9
+B01, B03	77.9	75.2	3.0	8.26	121	-0.04	82.6	+5.3	51.4
+B02, B03	78.4	73.0	2.8	9.0	111	+0.7	83.4	+6.1	50.8
Our method	81.1	75.6	3.2	8.6	117	+0.3	84.8	+7.5	50.2

1. The parameter count of our method has increased by 0.6M from YOLO11’s 2.6M, in line with YOLOv8n’s parameters. Thus, the model size is still reasonable.
2. Incorporating BERT does not increase depth and params. However, the inclusion of BERT extends overall runtime, primarily slowing down model training. Although the model’s accuracy (+B02) did not show a significant improvement over the baseline, it effectively enhances overall detection capabilities when combined with other modules, demonstrating a synergistic effect. This indicates that the semantic priors (environmental context) can enhance robustness under challenging conditions (occlusion, lighting variations).

To validate the stability and statistical reliability of the model’s performance improvement, we conducted separate training runs with five distinct random seeds (355, 31, 1102, 859, and 57) under the same hardware settings and evaluated both the baseline and our method. **Figure 12** shows the curve of the mAP50 for each run, along with the mean and standard deviation. Both models exhibited stable training processes, indicating that the performance was not caused by random fluctuations.

**Figure 12:**
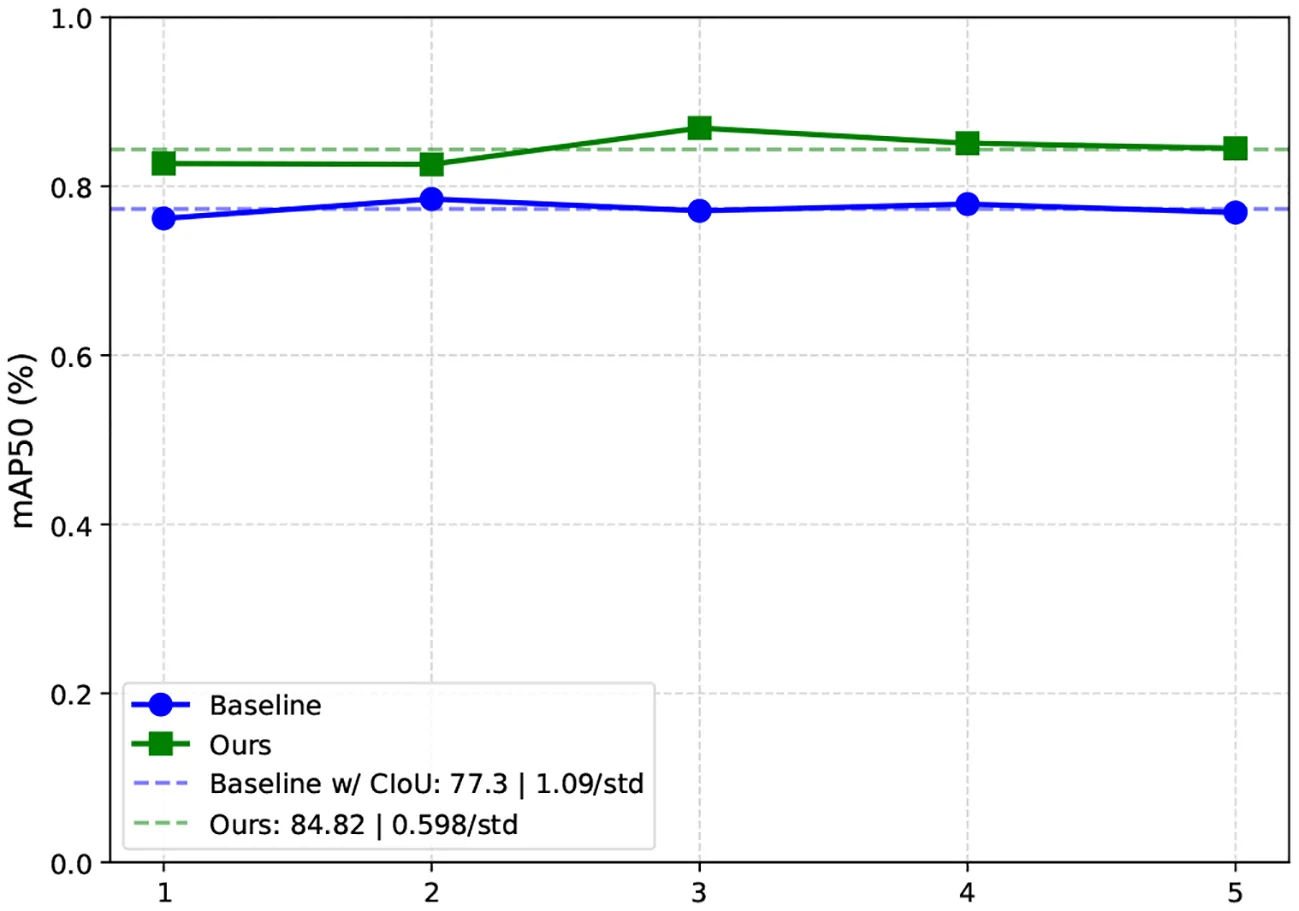
Stability analysis over five independent runs. The x-axis, #runs, is the run number with different random seeds.

### 4.6 Analysis of pepper flower detection results

To verify the visual improvements of our method over the baseline YOLO11, we intentionally chose two typical scenarios. The first features a high-brightness background where the colors visually resemble chili flowers, causing interference. The second involves a distant shooting with a chaotic background containing soil, flower pots, and other objects, where chili flowers occupy a small portion of the image. Both scenarios contain numerous chili flowers, falling under the category of multi-object detection.

In the 1st scenario, due to the relatively dim overall lighting conditions, the bright white patches in the background, which resemble the color of chili flowers, have a certain impact on the models. The main objects include 22 chili flowers, 3 green chilies, and 2 chili flower buds. Although the chili peppers and flower buds are not actually targets, they might still be misclassified as chili flowers during recognition. Therefore, they are listed as such. Both the flower buds and the chili peppers are misclassified for the automated pollination task. The overall model recognition results are summarized as shown in **Table 8**.

**Table 8:** Detection performance in scenario 1.

Model	#Corr.		#Misc.(from)				#conf<0.5 & missed
	Prec.	Part.	Flower bud	Pepper fruit	Background	Other	
YOLOv8	8	1	1	–	–	–	13
Baseline	9	1	–	–	1	–	12
YOLOv12n	5	4	–	–	1	–	13
RT-DETR	9	1	–	–	1	–	12
Faster R-CNN(ResNet50-FPN)	5	3	–	–	–	–	14
Our method	10	1	–	1	–	1	11

“Prec.” means the prediction box aligns almost perfectly with the GT box. “Part.” means the prediction box is accurately positioned but only partially encloses the object.

Columns #Corr. and #Misclassified (Misc.) in the table represent samples with a confidence *>* 0.5, corresponding to **Figure 13**. The last column #conf*<* 0.5 & Missed includes samples with a confidence *<* 0.5, and those were not identified as all. The experimental results show that both flower buds, pepper fruit, branches, and the background can be misclassified as chili flowers. These elements all serve as interference factors. However, in this scenario, our method outperforms other models in terms of the number of correctly identified chili flowers, with an accuracy rate higher than that of other models, demonstrating that our method is more robust to interference than others.

**Figure 13:**
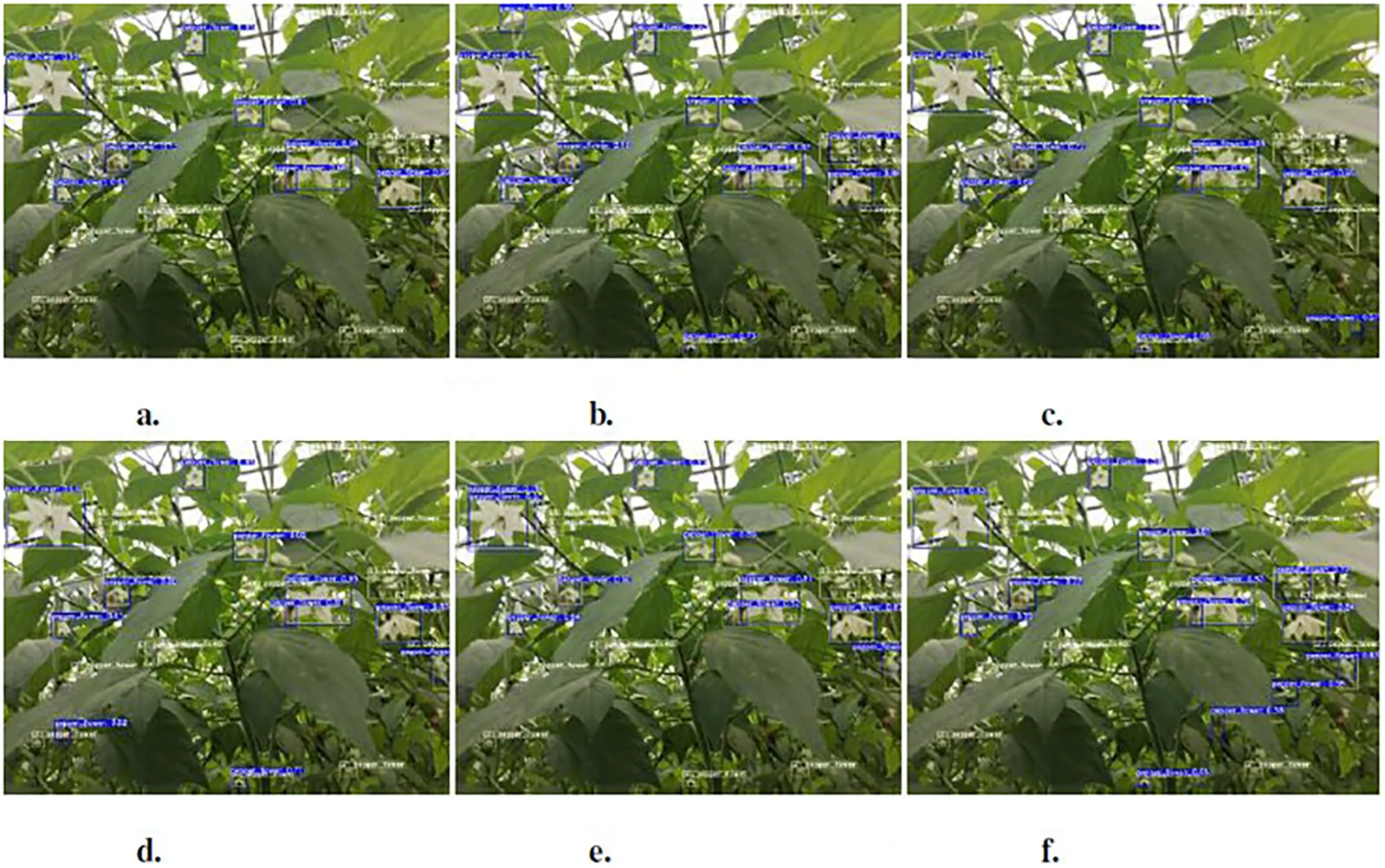
Chili flower recognition performance under different backgrounds.

In the 2nd scenario, the white granules in the flowerpot and the occlusion have a certain impact on performance. The main objects include 29 chili flowers and 8 bell peppers. Misclassifications occur for chili peppers, branches, leaves, and the background. The statistics for the experimental results in **Figure 14** are shown in **Table 9**.

**Figure 14:**
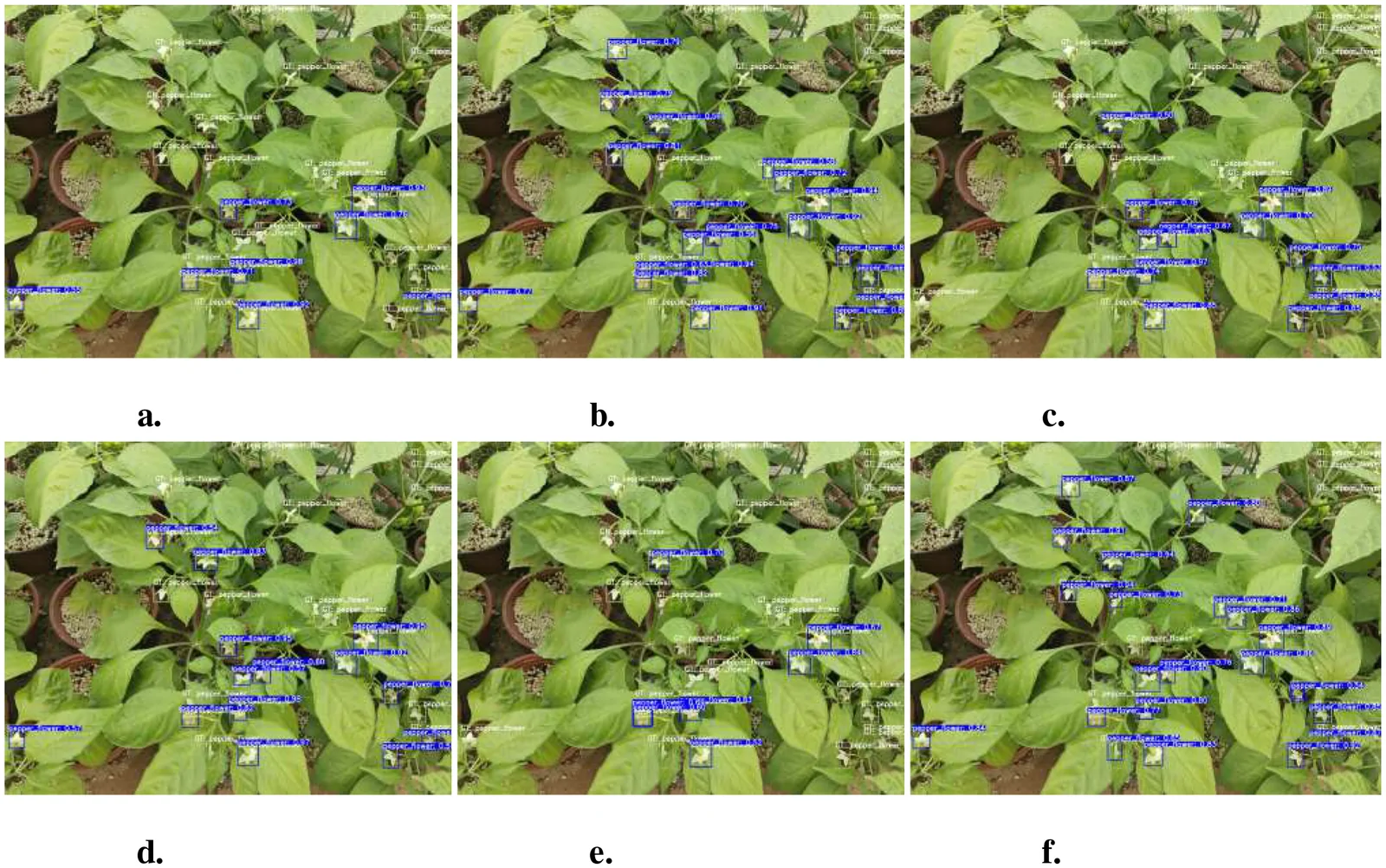
Chili flower recognition performance under different backgrounds.

**Table 9:** Detection performance in scenario 2.

Model	#Corr.		#Misc.	#conf<0.5 & missed
	Prec.	Part.		
YOLOv8	11	2	–	16
Baseline	11	3	–	15
YOLO12	6	1	–	22
RTDETR	16	4	–	9
Faster R-CNN(ResNet50-FPN)	7	1	–	21
Our method	18	3	–	8

Columns #Corr. and #Misc. present statistics for samples with a confidence *>* 0.5. Since there are no misclassifications in this scenario, column #Misc. is not broken down in detail. Like scenario 1, column #conf*<* 0.5 & Missed shows the total number of samples with a confidence *<* 0.5 and those that were not recognized. In terms of the number of correctly recognized instances (confidence *>* 0.5), our method outperforms other models.

**Figures 13**, **14** demonstrates that in both scenarios, our method achieves significantly higher detection counts than YOLO11, as well as other models, at confidence exceeding 0.5. Regarding confidence scores, YOLO11 exhibits higher values for the same objects in **Figures 13d, f** compared to our method. This indicates that deeper networks facilitate learning more complex structures, thereby boosting detection rates. This situation improved in **Figures 14d, f**, where our method not only outperformed YOLO11 in detection counts but also achieved higher confidence for several objects. In conclusion, our method displays superior performance for recognizing chili flowers in greenhouses, overall surpassing YOLO11.

## 5 Conclusion and discussion

Greenhouses represent a classic setting in modern agriculture, serving as a main battleground for smart agriculture due to their controllable environments. However, the complexity of conditions, as well as high crop density, pose persistent challenges for efficient, precise object detection. Chili peppers, a popular greenhouse cash crop, have long been considered inaccessible for yield enhancement through artificial pollination, primarily due to their tiny, densely clustered flowers. Automatic pollination requires improved detection accuracy. Building on YOLO11, this paper presents three strategies: Full shuffle overcomes the drawback of channel shuffle, where information exchange is incomplete when shuffling based on the entire channel position. By dividing feature maps into regions, full shuffle achieves complete information exchange while minimizing complexity, significantly improving model performance. The text-described external conditions enter the processing workflow via TinyBert encoding, enriching the feature maps and enhancing robustness against environment changes. Semantic priors prevent overfitting to spurious correlations through spatial regularization in full shuffle and refined localization in improved powerful IoU, thereby achieving precise spatial coordinate mapping. Unlike the CIoU, which includes computationally intensive aspect ratio, our variant significantly reduces computational burden while preserving the bounding box localization accuracy advantages of powerful IoU, enhancing flexibility—a critical consideration in resource-limited agricultural applications.

We acknowledge the following limitations regarding the dataset and the scope of the experiment: All images were taken in a single show hall in Shouguang, Shandong. Environmental factors (lighting, humidity) may differ from those in other greenhouses or open fields, potentially influencing model generality. This dataset primarily consists of fully bloomed flowers. Early buds or senescent flowers have not been labeled. Performance on those stages remains untested. This dataset covers only one kind of chili pepper. Flowers from different types may differ in color, size, and petal arrangement. Our model has not yet been verified on other varieties. While the dataset adequately illustrates the effectiveness of the proposed method under controlled conditions, its scope is limited. Therefore, results should be interpreted with caution when considering deployment of this method in different greenhouse environments, varieties, or imaging conditions. In addition, the dataset and data-splitting strategy might provide a risk of data leaks.

In future work, we plan to improve our method’s generalizability by collecting data from different locations, varieties, and seasons. Furthermore, we will systematically test our method on edge platforms to fully evaluate the model’s feasibility for deployment in commercial greenhouse environments.

## Data Availability

The raw data supporting the conclusions of this article will be made available by the authors, without undue reservation.
